# Circulating Betatrophin Is Strongly Increased in Pregnancy and Gestational Diabetes Mellitus

**DOI:** 10.1371/journal.pone.0136701

**Published:** 2015-09-01

**Authors:** Lana Kosi Trebotic, Peter Klimek, Anita Thomas, Anna Fenzl, Karoline Leitner, Stefanie Springer, Florian W. Kiefer, Alexandra Kautzky-Willer

**Affiliations:** 1 Department of Medicine III, Clinical Division of Endocrinology and Metabolism, Gender Medicine Unit, Medical University of Vienna, Vienna, Austria; 2 Clinical Division of Gynaecology and Obstretics, Medical University of Vienna, Vienna, Austria; 3 Section for Science of Complex Systems, CEMSIIS, Medical University of Vienna, Spitalgasse 23, Vienna, Austria; University of Oxford, UNITED KINGDOM

## Abstract

**Aims/hypothesis:**

Betatrophin has recently been introduced as a novel hormone and promotor of beta cell proliferation and improved glucose tolerance in mouse models of insulin resistance. In obese and diabetic humans altered levels were reported and a role in pathophysiology of metabolic diseases was therefore hypothesized. However its release and regulation in women with gestational diabetes mellitus (GDM), as well as its associations with markers of obesity, glucose and lipid metabolism during pregnancy still remain unclear.

**Methods:**

Circulating betatrophin was quantified in 21 women with GDM and 19 pregnant body mass index-matched women with normal glucose tolerance (NGT) as well as 10 healthy age-matched non-pregnant women by enzyme-linked immunosorbent assay. Additionally we performed radioimmunassay (RIA) to confirm the results.

**Results:**

Betatrophin concentrations measured by ELISA were significantly higher in GDM than in NGT (29.3±4.4 ng/ml vs. 18.1±8.7 ng/ml, p<0.001) which was confirmed by RIA. Betatrophin did not correlate with BMI or insulin resistance but showed a weak association with leptin levels in pregnancy and negative relationship with fasting C-peptide levels in all women. Moreover it correlated significantly with lipid parameters including triglycerides and total cholesterol in pregnancy, as well as estrogen, progesteron and birth weight.

**Conclusions/interpretation:**

Circulating betatrophin concentrations are dramatically increased in pregnancy and are significantly higher in GDM versus pregnant NGT. In the light of the previously reported role in lipid metabolism, betatrophin may represent a novel endocrine regulator of lipid alterations in pregnancy. However additional studies are needed to elucidate whether hormonal factors, such as estrogen, control the production of betatrophin and if targeting betatrophin could hold promise in the fight against metabolic disease.

## Introduction

Gestational diabetes mellitus (GDM) affects up to 8% of all pregnancies and is one of the most common gestational complications.[1] GDM is associated with an increased maternal incidence of type 2 diabetes mellitus, metabolic syndrome and cardiovascular disease at follow-up as well as various adverse acute and long-term outcomes among the offspring.[2, 3] Betatrophin also known as lipasin, refeeding-induced fat and liver (RIFL), hepatocellular carcinoma associated protein TD26 and angiopoietin-like protein 8 (ANGPTL8), is a recently discovered hormone that is primarily secreted by the liver but also by adipose tissue.[4] Its expression was increased in insulin resistant mice and overexpression promoted pancreatic β cell proliferation and improved glucose tolerance. [5] Based on these results, betatrophin has now been suggested as a novel candidate for therapeutic approaches involving beta cell regeneration in diabetes.[6, 7] Recent studies in humans have revealed conflicting results with regard to betatrophin regulation in obesity and diabetes. Whereas some studies showed increased circulating concentrations in obese or diabetic subjects, others found the opposite or no difference.[8–10] As underlying reasons, methodological issues have been discussed, because of potential betatrophin cleavage products in the plasma. The beneficial effects of betatrophin on beta cell growth and insulin secretion seen in one mouse study were not reproducible in human beta cells.[11] Hence to date the role of betatrophin in human beta cell function and its pathomechanistic relevance for diabetes remains enigmatic. Previous animal studies have suggested an important role for betatrophin in lipid, particularly in triglyceride metabolism. Genetic ANGPTL8 deficiency resulted in significantly lower triglyceride levels in response to refeeding whereas no alterations in glucose homeostasis were found. Accordingly, adenovirus-mediated overexpression of betatrophin doubled plasma triglyceride levels in mice without affecting beta cell proliferation.[12] Betatrophin has been demonstrated to affect blood lipid profiles by mechanisms involving regulation of hepatic very low density lipoprotein (VLDL) secretion as well as altered lipoprotein lipase (LPL) activity [4, 13]. Betatrophin may also act in concert with other angiopoietin-like protein family members such as ANGPTL3 [4, 13], a known regulator of cholesterol metabolism in mice and humans [14, 15]. Our own group recently reported a strong association between circulating betatrophin concentrations and atherogenic lipid parameters in obese and type 2 diabetic subjects, further supporting a potential role of betatrophin in lipid metabolism.

Pregnancy is not only a state of insulin resistance and physiological beta cell hyperplasia but also a condition characterized by dramatic alterations in plasma lipid profiles.

In GDM the combination of enhanced insulin resistance, impaired insulin release, and pregnancy-related hormonal changes contribute to an unfavorable dyslipidemic state.[16] Also a positive correlation between maternal triglycerides (TG) and neonatal body weight has been detected.[17] Overall these findings suggest that altered maternal lipid metabolism rather than hyperglycaemia constitutes a risk for diabetic fetopathy and macrosomia in GDM.[18] Release and regulation of betatrophin in women with GDM as well as its association with glucose and lipid metabolism have not been elucidated yet. Therefore, the aim of our study was to investigate the role of betatrophin in subjects with GDM and its association with lipid and glucose metabolism.

## Patients and Methods

Our study complies with the Declaration of Helsinki and Good Clinical Practice. Plasma samples were obtained from participants of studies previously approved by the human Ethics Committee of the Medical University of Vienna (EK-Nr. 1089/2009 and EK-Nr. 1123/2010). Both protocols contain paragraphs where is stated that plasma samples will be obtained and stored for investigation of future new biomarkers and adipokines. All subjects gave their written informed consent.

### Study Population

40 pregnant subjects were included in the study. 21 were newly diagnosed with GDM by a standardised 2-h-75-g-oral glucose tolerance test (OGTT) between gestational weeks 24 and 28 according to national and the IADPSG criteria.[19] The NGT group consisted of 19 BMI- matched healthy pregnant women, all without history of prior GDM or other comorbidities or medication intake.

The control group (CON) consisted of 10 healthy lean non-pregnant age-matched females with no previous history of pregnancy or diabetes. All study participants were healthy women, with the exception of the GDM group with diagnosed GDM. They had no history of any comorbidity or drug intake including lipid-lowering medication prior or during pregnancy except of those GDM women who needed insulin therapy for metabolic control during third trimester (N = 5). 3 patients in the GDM and 4 in the NGT group were multipara without history of prior GDM.

### Blood sample collection

Bloods samples were collected at the first visit at our patient clinic after overnight fasting. Serum and plasma parameters were determined at the Department of Medical and Chemical Laboratory Diagnostics of the Medical University of Vienna according to routine procedures. Homeostasis model assessment-estimated insulin resistance (HOMA-IR) was calculated by previously validated formula [20]. Plasma betatrophin concentrations were quantified using an ELISA kit (Wuhan Eiaab Science, Wuhan, China, Catalog No: E11644h) as previously described.[10]

As a second confirmatory readout we analyzed betatrophin concentrations in the same plasma samples using a commercially available RIA kit (Catalog Nr.: RK-051-60 by Phoenix Pharmaceuticals, Inc.) according to manufacturer’s instructions.

### Statistical Analysis

Comparisons of quantitative variables among groups were performed by one way ANOVA with post hoc Tukey’s test, whereas Kruskal-Wallis test with post hoc Mann Whitney analysis was used for not normally distributed data. Correlation analysis between continuous variables was performed by Pearson`s (normally distributed) or by Spearman`s analysis (not normally distributed data). Partial rank correlation was used for not normally distributed data. Data are given as mean ± SD. Levels of statistical significance were set at p<0.05. All tests were performed using SPSS Version 16.0, SPSS Inc., Chicago, IL. In addition a multivariable linear regression was performed to further corroborate that the association between GDM and betatrophin was not confounded by another variable. The regression was performed using MatLab’s built-in function using the robust fitting option in order to exclude the possibility that our results are driven by single, extremely small or large parameter values. The multivariable linear regressions were performed with betatrophin as response variable and the remaining patient characteristics that are shown in Table 1 as predictor variables. Birth weight was excluded from this analysis due to missing data (values were only available for 27 out of 40 patients). A categorical variable was added that depicts whether the patient belongs to the GDM, NGT, or CON group. In the first regression model only patients from group GDM and NGT were used, whereas in the second model patients from CON were also included. In the first model GW and AUC Glucose could be included as predictor variable, as opposed to the second model.

**Table 1:** Baseline charateristics of the whole study group.

	GDM		NGT		CON		p-value 1 GDM vs NGT	p-value 2 GDM vs CON	p-value3 NGT vs CON
	**n**		**n**		**n**				
**Age (years)**	21	30.95±5.15^1^	19	34.53±4.23^1^	10	32.40±5.30	0.026	0.538	0.258
**Body-Mass-Index (kg/m** ^**2**^ **)**	21	29.57±5.62^1^	19	29.81±6.97^1^	10	21.78±1.99^1^	0.878	<0.001	<0.001
**Triglycerides (mg/dl)**	21	263.57±98.49	19	186.53±76.62	10	76.60±38.62^1^	0.005	<0.001	<0.001
**Total Cholesterol (mg/dl)**	21	274.05±62.88	19	234.26±45.33	10	195.20±28.80	0.047	0.001	0.136
**HDL-cholesterol (mg/dl)**	21	67.67±14.43	18	72.33±18.19	9	82.78±25.65	0.708	0.107	0.350
**LDL-cholesterol (mg/dl)**	21	157.49±50.64	18	124.33±39.80^1^	9	96.73±29.19^1^	0.054	0.002	0.106
**HbA1c (%)/(mmol/mol)**	21	5.52±0.37/37.00±4.00	19	5.15±0.29/33.00±3.20^1^	10	5.07±0.19/32±2.10	0.002	0.001	0.392
**Insulin (mU/l)**	21	7.72±5.77^1^	19	7.51±6.03^1^	5	6.18±3.52^1^	0.661	0.837	0.899
**C-peptide (nmol/l)**	21	2.56±0.82^1^	19	2.22±1.08^1^	4	2.05±1.08	0.217	0.223	0.829
**Leptin (ng/ml)**	21	85.54±22.96^1^	19	43.32±15.12^1^	9	18.63±10.69^1^	<0.001	<0.001	<0.001
**Fasting glucose (mg/dl)**	21	94.52±11.41	17	85.94±5.63^1^	10	88.20±9.03	0.001	0.127	0.419
**HOMA-IR**	21	1.83±1.46^1^	17	1.61±1.31^1^	5	1.33±0.87^1^	0.428	0.527	0.895
**HOMA-β**	21	98.67±68.42^1^	17	123.60±101.14^1^	5	104.71±35.54^1^	0.637	0.613	0.895
**Gestational week**	21	29.95±3.32	16	26.31±4.69	0	na	0.009	na	na
**AUC Glucose (mg min/dl)**	21	17404.29±2169.57	16	15215.63±1755.63^1^	0	na	0.002	na	na
**Estrogen (pg/ml)**	21	13513.49±3958.78	19	7918.53±5145.84^1^	6	77.33±85.83^1^	<0.001	<0.001	0.001
**Progesteron (ng/ml)**	21	134.87±49.17^1^	19	80.64±69.42^1^	3	3.36±4.72^1^	0.001	0.001	0.030
**Birth weight (g)**	13	3664.23±368.91	14	3185.36±380.72	0	na	0.003	na	na

Baseline charateristics of healthy controls (CON), women with gestational diabetes (GDM), and with normal glucose tolerance (NGT); Baseline data are given as mean±SD.

Normally distributed data were compared by one way ANOVA with post hoc Tukey test, whereas Kruskal-Wallis test with post hoc Mann Whitney analysis was used for not normally distributed data (^1^). Differences were considered statistically significant at exact 2-sided values of p<0.05.

## Results

40 pregnant women (21 with GDM and 19 NGT) in gestational weeks 24 up to 28, as well as 10 healthy lean age-matched female controls (CON), have been included in our study (Table 1).

**Fig 1:**
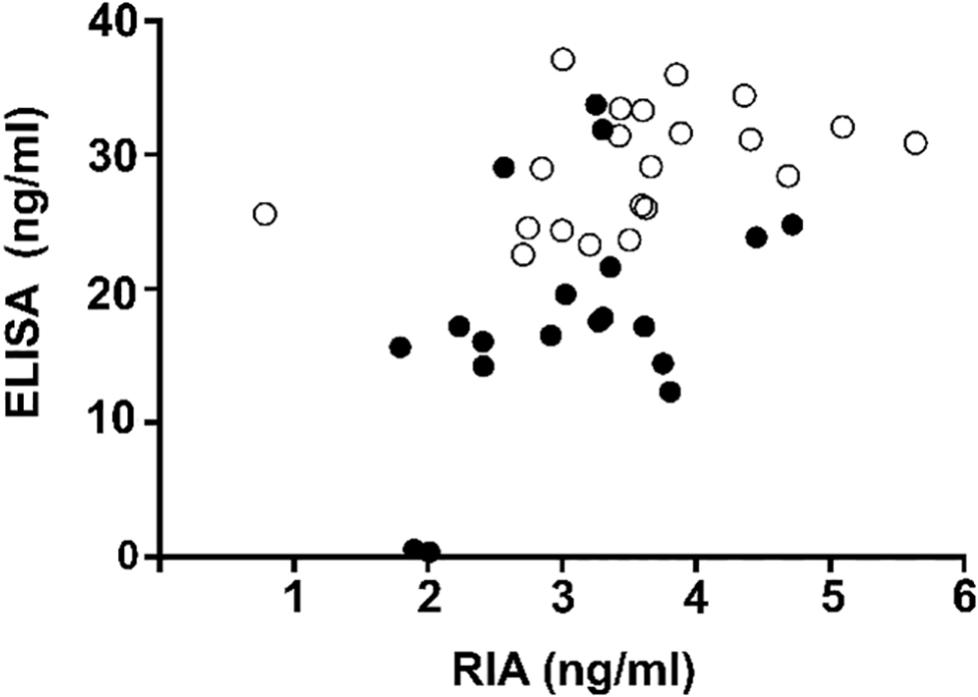
Correlation between Betatrophin ELISA and Betatrophin RIA data. Pearsons r = 0.760, p<0.001.

**Fig 2:**
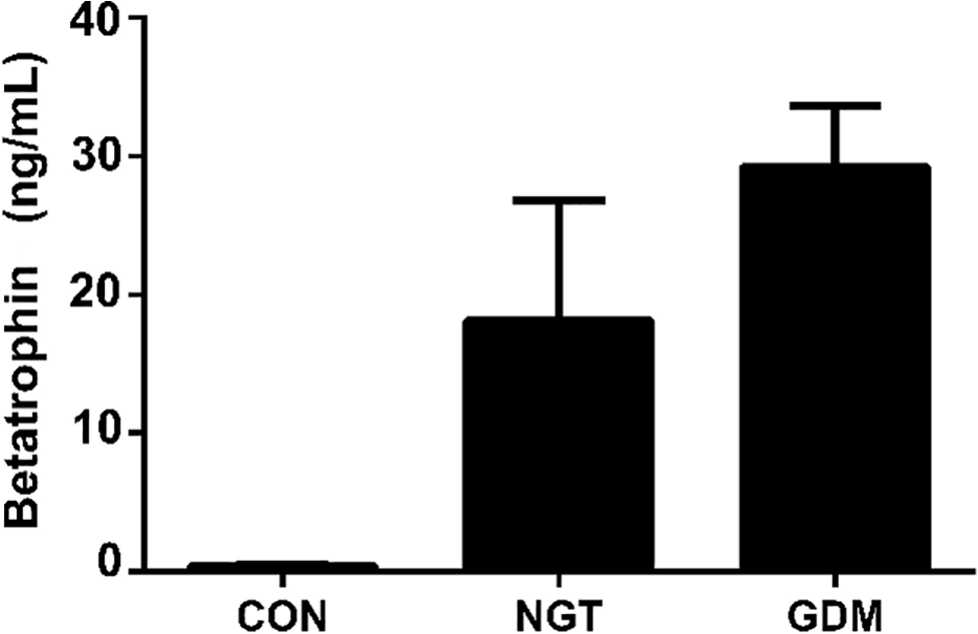
Betatrophin differences between groups. Values are expressed as mean±SD in ng/ml, GDM: 29.24±4.39, NGT: 18.12±8.65, CON: 0.42±0.13; GDM vs NGT p<0.001, GDM vs CON p<0.001, NGT vs CON p<0.001.

Betatrophin concentrations were determined in plasma samples by both, ELISA and RIA. In accordance with previously published findings the concentrations measured by RIA were markedly lower compared to ELISA; however the relative differences between the study groups remained similar. As shown in Fig 1, both assays correlated significantly with one another. Notably, betatrophin concentrations were dramatically higher in pregnant women (GDM and NGT) compared to non pregnant controls (Fig 2). Among pregnant women, GDM had significantly higher betatrophin levels than NGT (Fig 2).

We studied the correlations between circulating betatrophin and parameters of glucose homeostasis. Betatrophin was neither related to insulin or C-peptide concentrations nor to HOMA-IR, HOMA-ß and BMI. However in the NGT group we noticed a moderate negative correlation between betatrophin and fasting glucose as well as in pregnancy a weak positive correlation with HbA1c, suggesting no clear association between betatrophin levels and insulin resistance in pregnancy. Betatrophin concentrations correlated significantly with total cholesterol and triglycerides in pregnancy and CON (Table 2) whereas there was only a trend for a positive correlation with LDL cholesterol (p = 0.064).

**Table 2:** Correlations between betatrophin and metabolic parameters.

		GDM	NGT	GDM+NGT	CON
**Age**	r:	0.268^1^	0.058^1^	-0.150^1^	0.466
	p:	0.241	0.814	0.355	0.174
**BMI**	r:	-0.209^1^	0.335^1^	0.073^1^	-0.049^1^
	p:	0.363	0.161	0.657	0.894
**Fasting Glucose**	r:	-0.002	-0.600^1^	0.123	0.000
	p:	0.993	0.011	0.461	1.000
**HbA1c**	r:	-0.009	0.145^1^	0.363	0.054
	p:	0.970	0.553	0.022	0.882
**Insulin**	r:	-0.277^1^	-0.105^1^	-0.102^1^	0.000^1^
	p:	0.224	0.669	0.532	1.000
**C-peptide**	r:	-0.272^1^	-0.104^1^	-0.060	-0.133
	p:	0.234	0.670	0.711	0.867
**Leptin**	r:	-0.523^1^	-0.060^1^	0.402^1^	-0.055^1^
	p:	0.015	0.808	0.010	0.881
**HOMA-IR**	r:	-0.300^1^	-0.201^1^	-0.093^1^	0.000^1^
	p:	0.186	0.439	0.577	1.000
**HOMA-β**	r:	-0.365^1^	0.088^1^	-0.150^1^	-0.100^1^
	p:	0.104	0.736	0.369	0.873
**AUCgluc.**	r:	-0.444	0.088^1^	0.253	na
	p:	0.044	0.745	0.131	na
**Estrogen**	r:	0.545	0.698^1^	0.733	0.086^1^
	p:	0.011	0.001	<0.001	0.872
**Progesterone**	r:	0.282	0.702^1^	0.617	-0.500^1^
	p:	0.216	0.001	<0.001	0.667
**TG**	r:	0.248	0.527	0.520	0.638^1^
	p:	0.278	0.020	0.001	0.047
**Tot. Chol.**	r:	0.253	0.323	0.409	0.640
	p:	0.268	0.177	0.009	0.046
**LDL-C.**	r:	0.243	-0.042^1^	0.300	0.367^1^
	p:	0.289	0.868	0.064	0.332
**HDL-C.**	r:	0.122	0.406	0.120	0.017
	p:	0.599	0.095	0.466	0.965
**Birth weight**	r:	0.175	0.418	0.658^1^	na
	p:	0.568	0.137	<0.001	na

healthy controls (CON), women with gestational diabetes (GDM), and with normal glucose tolerance (NGT)

Correlation analysis between continuous variables was performed by Pearson`s (normally distributed) or by Spearman`s analysis (not normally distributed data (^1^)) with r representing correlation coefficient and p the statistical significance. Differences were considered statistically significant at exact 2-sided values of p<0.05

Estrogen correlated significantly with betatrophin in NGT and GDM but not in CON (Table 2), Also progesterone correlated with betatrophin levels in the NGT group and in the combined group of NGT and GDM but not in GDM alone or in CON. The birth weight of the babies from the GDM group was significantly higher than from the NGT group (p = 0.003), and strongly correlated with maternal betatrophin concentrations (Spearmans r = 0.658, p<0.001).

This correlation remained significant after adjusting for TG (partial rank correlation r = 0.565, p = 0.003), estrogen (partial rank correlation r = 0.396, p = 0.045), or total cholesterol (partial rank correlation r = 0.586, p = 0.002).

Results of the regression models are shown in Table 3, where estimates of the coefficients for the two models and the p-values to reject the null hypotheses that the true values of the coefficients are zero are shown. In both models the predictor variable with the highest effect on betatrophin is GDM. In model 1 using patient groups GDM and NGT it is also the only significant predictor variable. In model 2 betatrophin is positively related with estrogen and BMI, whereas C-peptide levels are negatively related with betatrophin. All other estimates of coefficients are not significantly different from zero. This corroborates the result that betatrophin concentrations were significantly higher in GDM than in NGT patients, also controlling for other influences such as BMI or insulin resistance.

**Table 3:** Multivariable linear regressions.

Parameter	Model 1 (GDM+NGT)	Model 2 (GDM+NGT+CON)
**(Intercept)**	-7.2(31) 10^3^	-2.9(2.2) 10^4^
	*p* = 0.82	*p* = 0.20
**Age (years)**	-13(310)	104(290)
	*p* = 0.97	*p* = 0.72
**Body-Mass-Index (kg/m** ^**2**^ **)**	320(250)	**420(200)**
	*p* = 0.22	***p* = 0.048**
**Triglycerides (mg/dl)**	27(24)	32(22)
	*p* = 0.27	*p* = 0.16
**Total Cholesterol (mg/dl)**	-220(150)	-170(130)
	*p* = 0.15	*p* = 0.18
**HDL-cholesterol (mg/dl)**	200(170)	160(130)
	*p* = 0.26	*p* = 0.24
**LDL-cholesterol (mg/dl)**	200(140)	150(120)
	*p* = 0.19	*p* = 0.24
**HbA1c (%)/(mmol/mol)**	4800(3400)	6000(2900)
	*p* = 0.17	*p* = 0.050
**Insulin (mU/l)**	6400(3900)	5500(3700)
	*p* = 0.12	*p* = 0.16
**C-peptide (nmol/l)**	-3800(1900)	**-4800(1800)**
	*p* = 0.070	***p* = 0.012**
**Fasting glucose (mg/dl)**	40(170)	60(160)
	*p* = 0.82	*p* = 0.72
**HOMA-IR**	-2.3(1.4) 10^4^	-1.9(1.3) 10^4^
	*p* = 0.11	*p* = 0.15
**HOMA-β**	-62(59)	-56(56)
	*p* = 0.31	*p* = 0.33
**Gestational week**	220(390)	—
	*p* = 0.57	
**AUC Glucose (mg min/dl)**	-0.60(66)	*—*
	*p* = 0.37	
**Estrogen (pg/ml)**	0.64(36)	**0.85(0.31)**
	*p* = 0.093	***p* = 0.011**
**Progesteron (ng/ml)**	15(31)	21(28)
	*p* = 0.63	*p* = 0.45
**Birth weight (g)**	*—*	*—*
	
**Leptin**	-56(51)	-45(48)
	*p* = 0.29	*p* = 0.36
**GDM**	**1.3(4) 10** ^**4**^	**1.0(4) 10** ^**4**^
	***p* = 0.0094**	***p* = 0.0092**
**CON**	*—*	-1300(4500)
		*p* = 0.77
**R** ^**2**^	0.79	0.89

Results of two multivariable linear regressions of the response variable betatrophin are shown for the patient groups GDM and NGT, model 1, and GDM, NGT and CON, model 2. For each predictor variable we show its coefficient values and their standard errors in the respective models, together with the p-value to reject the null hypothesis that the true coefficient value is zero. We find that the presence of GDM has the strongest relation to increased betatrophin in both models. Whereas in model 1 GDM is the only predictor variable with a coefficient value that is significantly different from zero, betatrophin shows also an inverse relation with c-peptides and a positive relation with estrogen and BMI in model 2.

## Discussion

Betatrophin is a liver and fat derived hormone with implications in the regulation of lipid metabolism and, as proposed more recently, possibly in beta cell replication and glucose homeostasis. Human studies attempting to determine the role of betatrophin in various models of obesity, insulin resistance, and type 2 diabetes have delivered conflicting results. Hence, additional studies are critically needed to better understand the (patho) physiologic importance of this molecule, particularly in the context of metabolic disorders. In a recent preclinical study, hepatic betatrophin expression was not only increased in mouse models of insulin resistance but rose significantly during pregnancy which led the authors to the conclusion that betatrophin may contribute to beta cell expansion during gestation.

Here we demonstrate that circulating betatrophin is dramatically upregulated in pregnant versus non-pregnant women. Notably, betatrophin levels were even further increased in women with GDM. However, with the exception of fasting glucose in the NGT group, no association was found between betatrophin and parameters of glucose homeostasis in any of the tested groups which is in line with several previous reports.[10, 21] Our data also question whether the pregnancy-induced betatrophin increase is indeed related to altered beta cell mass. This contrasts the findings of a recent cross-sectional study in GDM where betatrophin was positively associated with FPG and HOMA-IR but inversely correlated with HOMA-β.[22] In the regression analysis we find that C-peptide-peptide levels are negatively related to betatrophin which is concordant with findings of Tokumoto et al. in non-pregnant subjects [22, 23] However in our study HOMA-ß did not correlate with betatrophin, but this calculated parameter is indeed not the most suitable parameter for evaluation of beta cell function. More sophisticated readouts of beta cell function would be required to fully address this issue. Also the fact that fasting insulin and c-peptide concentrations were not significantly different between groups limits the interpretation of this particular aspect.

The fact that leptin was the highest in GDM group suggests a state of leptin resistance as previously described.[24] This confirms previous findings where leptin was increased in GDM and showed a clear association with glucose concentrations and insulin resistance suggesting GDM as a state of insulin and leptin resistance. A recent meta-analysis on eight prospective studies on leptin has indicated high leptin concentrations as a risk factor for later development of GDM.[25]. Increased leptin in GDM also appears to reflect the increased amount of body fat stores. Of note we did not see a relationship between betatrophin and BMI in our study. Although leptin shows no significant influence on betatrophin in the regression analyses, we find a significant association between these variables in the correlation analysis. This suggests that the observed correlation between leptin and betatrophin can be fully explained by another variable, such as GDM.

Lipid metabolism undergoes significant changes during pregnancy mainly driven by adipose tissue accumulation followed by increased lipolysis and hyperlipidemia. This physiological hypercholesterolaemia has been attributed to the hormonal effects of progesterone and oestrogen.[26] The daily production of progesterone increases thirtyfold, while that of oestrogen increases tenfold during pregnancy. Progesterone increases plasma levels of LDL cholesterol and total cholesterol and lowers HDL cholesterol while oestrogen has an opposite effect.[27]

In contrast to two recent studies, that investigated the relationship between betatrophin and lipids, we found that betatrophin correlated with circulating lipid parameters such as triglycerides, total cholesterol, but not HDL cholesterol, in NGT and GDM women together, which is in accordance with our own previous findings in obesity and type 2 diabetes and other studies.[10, 28] Indeed betatrophin has been implicated in triglyceride regulation by mechanisms involving altered hepatic very low density lipoprotein secretion and lipoprotein lipase activity. Correlations between betatrophin and lipid parameters were not statistically significant in each group individually which might be due to the relatively small sample size.

Together our data could argue for a mechanistic role of betatrophin in lipid profile alterations during pregnancy and therefore warrant further investigation.

Interestingly betatrophin also correlated with estrogen and progesteron levels, raising the possibility of a positive regulation of betatrophin production and thus may contribute to the remarkable betatrophin elevation during pregnancy. However, detailed mechanistic studies are needed to further address this hypothesis.

As previously described, TG of the mother correlate with neonatal body weight.[16] We also found a moderate correlation between betatrophin and birth weight confirming the role of betatrophin in lipid homeostasis. These correlations were slightly attenuated but remained significant after adjusting for TG.

We are aware that this study has some limitations. The sample size is rather small which may not allow the detection of weak associations between betatrophin and parameters of glucose homeostasis. Matching of the patients was complicated by clincial differences between women who were recruited consecutively at the time of the diagnostic OGTT performed in our department. Therefore we performed partial rank correlations and adjusted the correlations with betatrophin for age and gestational week. The main results remained consistent so we concluded the suboptimal matching does not alter the outcome of the study. Nonetheless, the study design was well suited to detect the robust correlations between betatrophin and blood lipids. Given that plasma parameters were only measured once during pregnancy, no conclusions can be drawn regarding potential dynamic changes during the course of gestation. A distinct functional role of betatrophin in pregnancy and in the progression of metabolic complications in GDM cannot be addressed using such a cross sectional study design. However, the reported results may further instigate the discussion about the pathophysiologic relevance of this molecule, particularly in lipid disorders. Future studies will have to address whether betatrophin represents a potential novel drug target for lipid-lowering treatment and/or a cardiovascular risk factor that may suggest close monitoring and post-partum follow up investigations, particularly in women with prior GDM who are known to be at increased cardiovascular risk.
